# Understanding estrogen receptor transcription in breast cancer

**DOI:** 10.1186/1868-7083-5-S1-S7

**Published:** 2013-08-19

**Authors:** Jason S Carroll

**Affiliations:** 1 Cancer Research UK, Cambridge Research Institute, Cambridge, CB2 0RE, UK

## 

Estrogen Receptor (ER) is the defining feature of luminal breast cancers, where is functions as a transcription factor. The traditional view of ER getting recruited to promoters of target genes is too simplistic. The recent discovery of ER-DNA interaction regions from ER+ breast cancer cell lines has revealed that ER rarely associates with promoter regions of target genes and instead associates with enhancer elements significant distances from the target genes. The genomic mapping of ER binding events also revealed the enrichment of DNA motifs for Forkhead factors. The Forkhead protein FOXA1 (HNF3a) was subsequently shown to bind to ~half of the ER binding events in the genome and was required for ER to maintain interaction with DNA. We have extended on these findings to map ER binding events in primary breast cancers and distant metastases. We find context dependent ER cis-regulatory elements (cistromes) that give insight into underlying transcriptional networks. These differential ER binding profiles correlate with clinical response in ER+ breast cancers. We experimentally explore the binding dynamics between drug sensitive and resistant contexts and identify properties that govern ER binding differences. These data suggest that ER-DNA interactions are dynamic and can be modulated by changes in FOXA1. We are currently exploring mechanisms that mediate FOXA1-DNA interactions, in order to better understand ER transcriptional activity in breast cancer biology.

